# Continuance of value

**Published:** 2015

**Authors:** VL Purcarea

**Affiliations:** * “Carol Davila” University of Medicine and Pharmacy, Bucharest, Romania

Romanian medicine has brought the world medicine a contribution that will last forever amongst universal values. George Emil Palade, Nicolae Constantin Paulescu, Ana Aslan, Stefan Odobleja, Thoma Ionescu, Sofia Ionescu, Dimitrie Bagdasar, Gheorghe Marinescu, Ion Cantacuzino, Victor Babes, Carol Davila, Matei Bals, C. C. Iliescu, Al. Trestioreanu, Marius Nasta, C. I. Parhon, N. Gh. Lupu, Mina Minovici, Al. Obregia, D. Hociota, P. Sarbu, Th. Burghele, Stefan S. Nicolau, Eugen Proca, etc., are only few of the Romanian personalities who decisively enriched the medical patrimony. Unfortunately, Academician Laurentiu Mircea Popescu, a brilliant representative of the world medical scientific research, joined them too early.

**Fig. 1:**
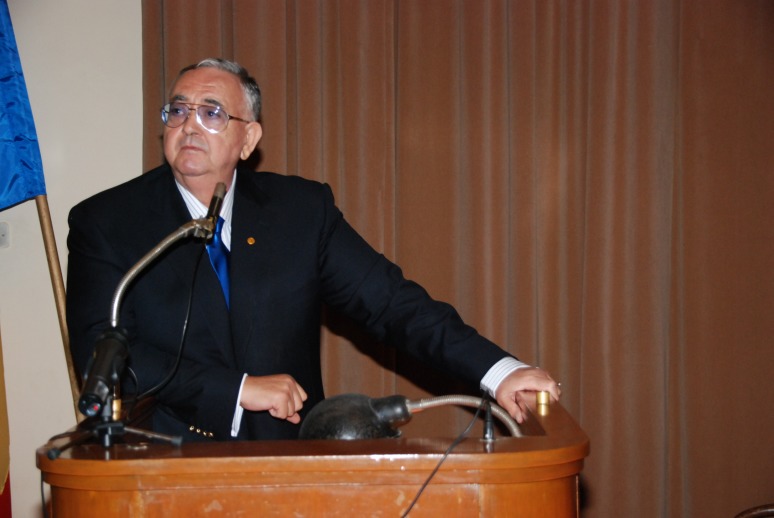
Acad. Laurentiu Mircea Popescu – the Opening of the University Year at “Carol Davila” University of Medicine and Pharmacy, Bucharest

He was born in Campulung, studied and formed in Bucharest, just like his destiny chose, on Prof. Dr. Gh. Marinescu Street, near “Carol Davila” University of Medicine and Pharmacy, which he graduated valedictorian in 1967 and, in which, few years later, in 1972, he became a medical doctor. Moreover, he was also a Teaching assistant (1971), Assistant professor (1979), Associate professor (1990), Professor (1992), Head of Department (1993), Head of specialty department (2001). He was also Vice-dean of “Carol Davila” University of Medicine and Pharmacy between 1990 and 1992 and Rector of “Carol Davila” University of Medicine and Pharmacy between 1992 and 2004. 

He was President of the Academy of Medical Sciences (2006-2011) and member of the Academy of Sciences in New York. He was also member of the American Society for Cell Biology, honor member of the Academy of Medicine in Poland, member of Albert Schweitzer International Academy in Switzerland, member of the International Academy of Cardiovascular Sciences in USA, honor member of the National Academy of Surgery in Paris, member of the Federation of European Academies of Medicine (FEAM). He was also President of the Department of Medical Sciences of the Romanian Academy (2006-2014) and general manager of “Victor Babes” National Institute in Bucharest (1993-2015). 

**Fig. 2:**
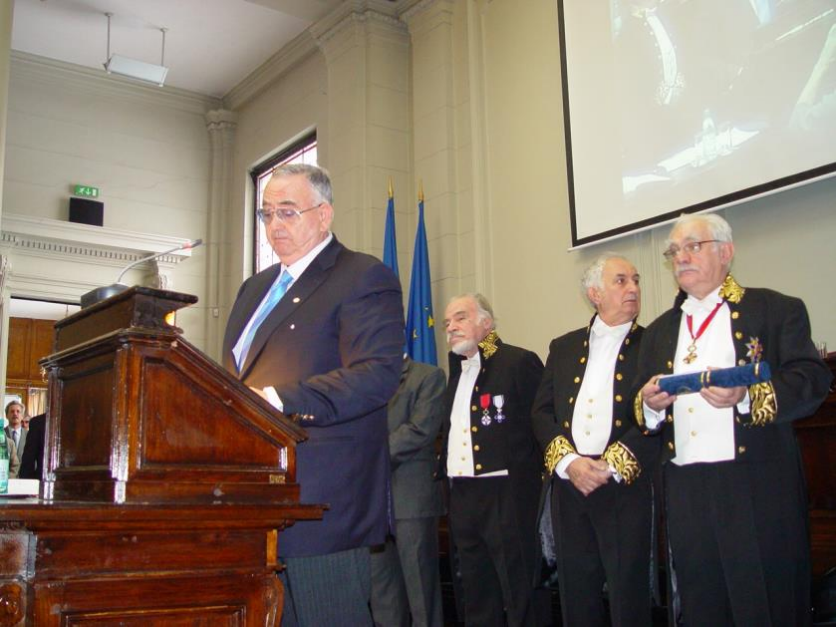
Acad. Laurentiu Mircea Popescu at the presidium of the Romanian Academy

**Fig. 3:**
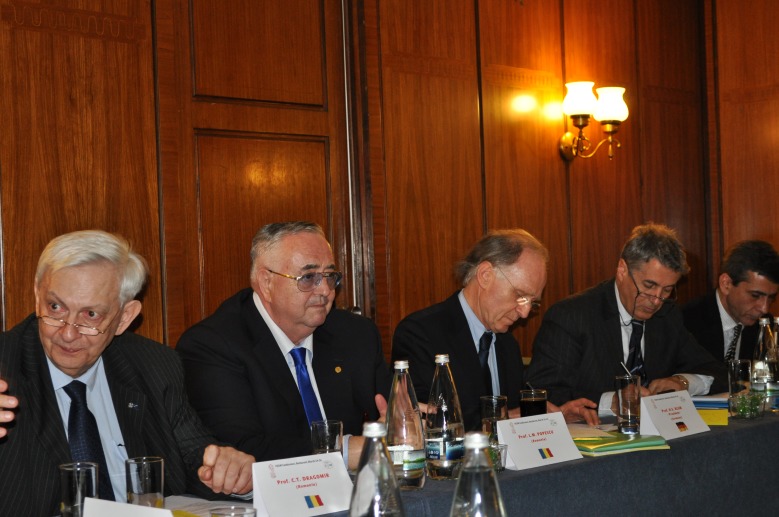
Acad. Laurentiu Mircea Popescu at the presidium of FEAM, in Bucharest

He had a sustained scientific activity, having published over 250 works and almost 300 communications (invited conferences, reports, and communications in different national or international events. Among the published papers, over 150 were published abroad in prestigious scientific journals such as Acta Histochemica et Cytochemica (Japan), American Journal of Physiology (USA), Annals of the New York Academy of Sciences (USA), Anatomical Records (USA), Applied Immunohistochemistry & Molecular Morphology (USA), Autophagy (USA), Biochemical and Biophysical Research Communications (USA), Biochemical Pharmacology (Belgium), Biochimica et Biophysica Acta (The Netherlands), BioMolecular Concepts (The Netherlands), Bioscience Reports (UK), Cell and Tissue Research (Germany), Cell Calcium (UK), Cellular Physiology and Biochemistry (USA), Cells Tissues Organs (Switzerland), Experimental Cell Research (Sweden), Histology and Histopathology (Spain), Journal of Neurology Sciences (Italy), Neuroscience Letters (The Netherlands), Acta Physiologica Hungarica (Hungary), etc.

He was also President of Foundation for Cellular and Molecular Medicine through which he sustained both “Carol Davila” University Press, while he was Rector of the University, and Journal of Cellular and Molecular Medicine from its beginning, being its Executive Editor even after it was taken by Blackwell Publishing. 

**Fig. 4:**
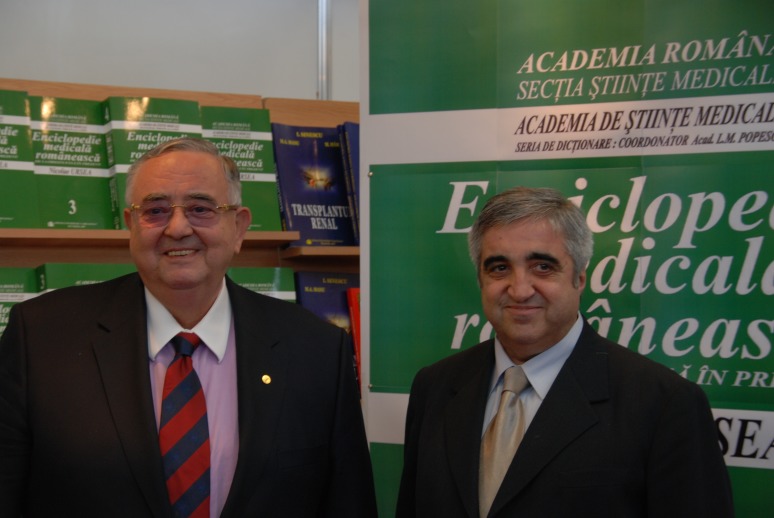
Acad. Laurentiu Mircea Popescu and Prof. Dr. Eng. Victor Lorin Purcarea, General Manager of “Carol Davila” University Press – launching the book “Medical Encyclopedia”

He was a member of the Editorial Board of the following journals: Acta Histochemica and Cytochemica (Japan), Cell Transplantation (USA), Chinese Journal of Clinicians (China), Comments on Molecular and Cellular Biophysics (The Netherlands), International Journal of Translational Medicine (USA), Italian Journal of Anatomy and Embriology (Italy), Langenbeck Archives of Surgery (Germany), World Journal of Stem Cells (China), World Journal of Methodology (China).

**Fig. 5:**
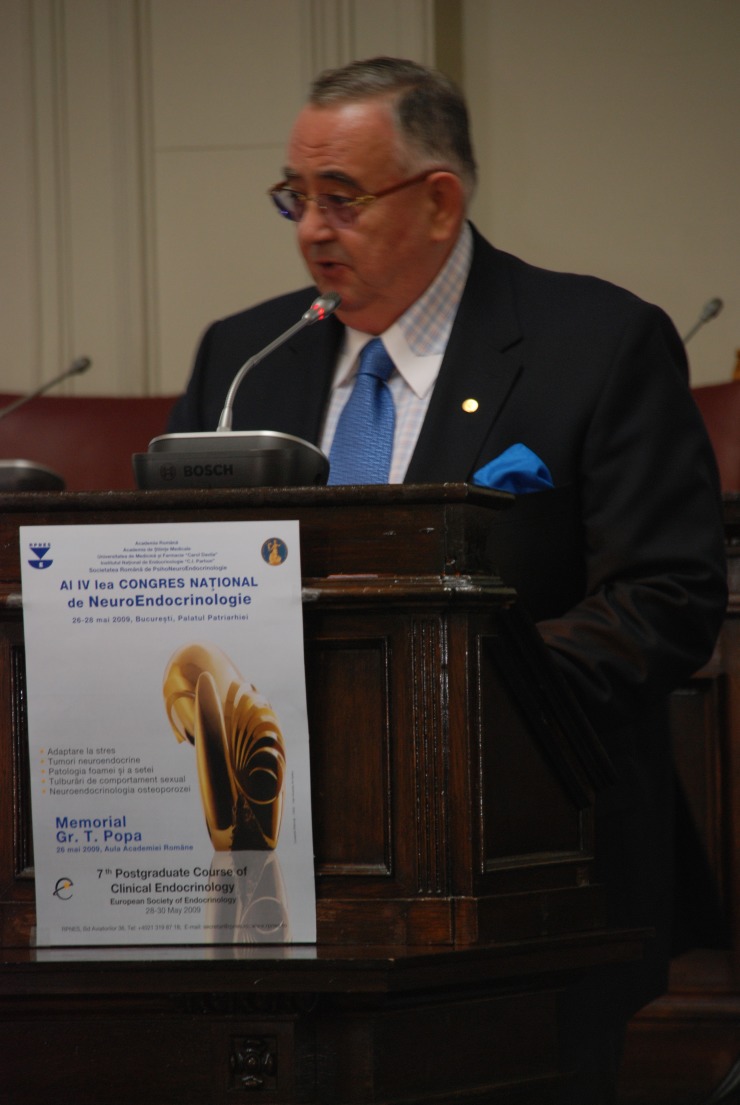
Acad. Laurentiu Mircea Popescu at the presidium of the 4th National Congress of Neuroendocrinology, Bucharest

He was awarded many honorary titles such as: Men of Achievement (England), 1986; Dictionary of International Biography (England), 1988; International Cultural Diploma of Honor (USA), 1995; Five Hundred Leader of Influence (USA), 1995; Millennium Hall of Fame (USA), 1998; Men of the Year (USA), 1999; International Man of the Millennium (USA), 2000; “100 Personalities in Health Sciences” - elected, Cambridge, 2010; having a Hirsch Index of 23 (according to Thomson ISI Database).

He was also awarded The Order of the “Romanian Star” as a commander in 2009 and “Victor Babes” Prize of the Romanian Academy in 1985. We was Senator in the Romanian Parliament between 2000 and 2004, he was a member of the Commission for Health, Ecology, Youth and Sport, but he was also the main member of the Commission of the Romanian Parliament in the Parliamentary Meeting of the Organization for Security and Cooperation in Europe. 

**Fig. 6:**
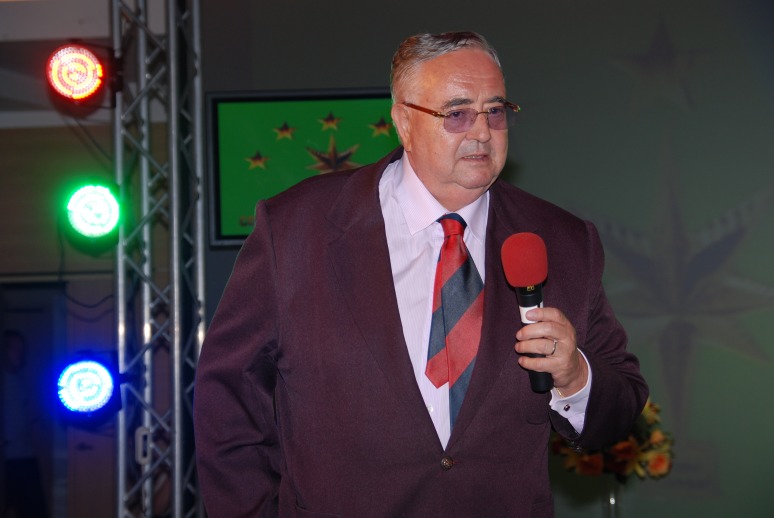
Acad. Laurentiu Mircea Popescu at FEAM Congress in Bucharest

**Fig. 7:**
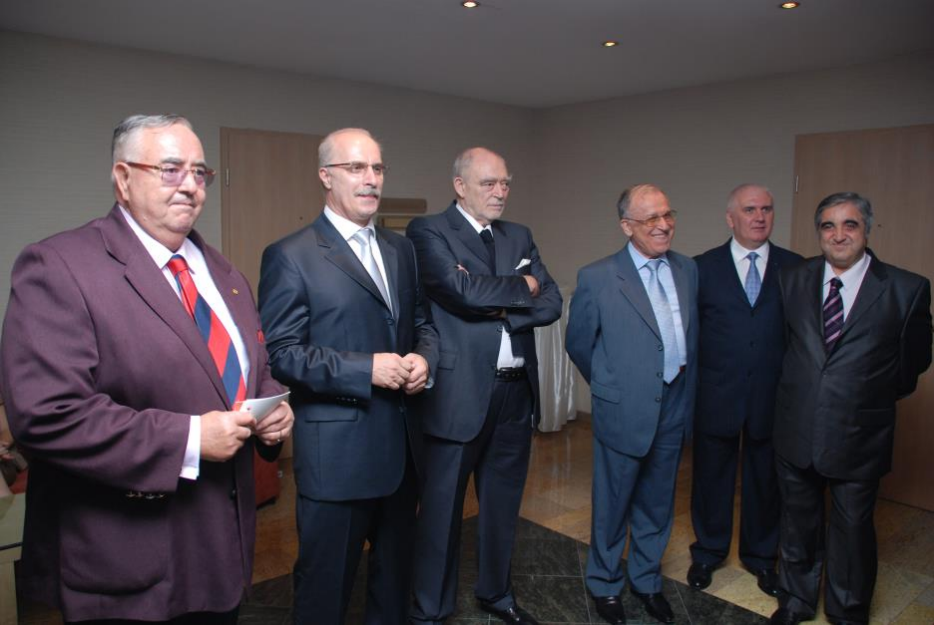
Acad. Laurentiu Mircea Popescu together with the former president of Romania and other guests at a reception

As a sign of appreciation of his valuable personality, 13 prestigious universities in the whole world offered him the title “Doctor Honoris Causa”. 

“Being a brilliant researcher, due to his profession and structure, and also being open to effort and to an unusual perceptiveness, Academician Laurentiu Mircea Popescu represents the most brilliant model in the Romanian Medical research field. Together with his Excellency, his team of researchers in “Victor Babes” National Institute of Pathology has discovered a new type of human cells – the telocytes, which were initially called “Popescu cells” or “Cajal-like” interstitial cells. These cells are seen abroad as the key to the tissue regeneration when combined with the stem cells. With their help, myocardial infarction, the main cause of death in the world, could be treated, declared Prof. Dafin Fior Muresanu, MD, at the beginning of 2015. 

The late academician was the first Romanian citizen who was offered the “Medal of Merit” in 2012 of the International Academy of Cardiovascular Sciences. This honor is considered a real Nobel Prize for cardiology, by the personalities in the international medicine. 

“It means a lot to me. To take it by far, this honor is shared with a professor in Harvard University, and, even if we share it, its moral, ideatic and scientific value will count the most. I think it is the most important honor of recognition of the scientific value in the last years, offered to a representative of the Romanian medical school and of the Bucharest medical school, of some traditions in “Victor Babes” Institute. It is, to my mind, an important step in taking the position Romanian research in cardiovascular field deserves on the map of Europe and of a significant part of the whole world”, declared on that occasion, with his well-known modesty, the famous scientist, in an interview offered to a prestigious journal. 

**Fig. 8:**
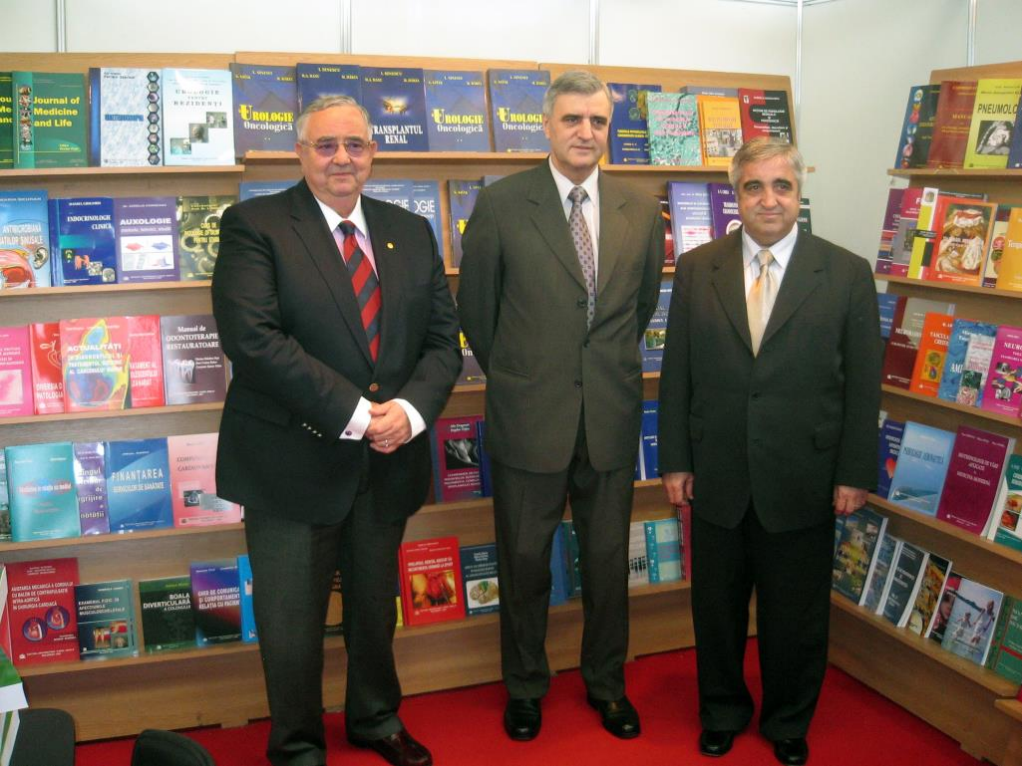
Acad. Laurentiu Mircea Popescu together with the Rector of “Carol Davila” University of Medicine and Pharmacy, Bucharest, Acad. Ioanel Sinescu, at the book stand of the University Press of “Carol Davila” University of Medicine and Pharmacy, at Rommedica

The importance of such an international appreciation was also recognized by important personalities in Romania. 

“It is extraordinary, the more so as we know that there are some difficulties in Romania as far as research is concerned. What is important is that perseverance in work, and, I could also say without exaggerating, the geniality in the research field, have all led to this discovery. I am telling you from the experience of my specialty that this only happens where there is a lot of recognition and appreciation for the work performed. In addition, it is a very special moment in our existence as academic, university, research members in Romania”, remarked Acad. Prof. Ioanel Sinescu, MD, Rector of “Carol Davila” University of Medicine and Pharmacy in Bucharest. 

The discovery of telocytes, an accidental matter, as a result of an endless perseverance and of an effort that cannot be measured, in looking for novelty, which were constantly developed in “Victor Babes” National Institute of Pathology, are confirmed now in at least 68 centers in Europe, in over 20 centers in USA and 10 centers in China. 

In “Confessions of a young novelist”, Umberto Eco wrote: “The fact is that we rarely define things according to their essence. We most often use lists of characteristics”. 

To quote one of the most serious and rigorous follower of the famous academician, the one who followed his footsteps and is at present Head of Department, Prof. Mihai Hinescu, “Umberto Eco’s statement can also be extended to the personalities in the scientific world. Academician Laurentiu Popescu had the rare ability of understanding and defining things in their essence. At the same time, he imposed his work, tenacity, and perseverance in completing “the lists of properties” of the cells and tissues, which he dealt with for the rest of all his life. 

He considered it very important to work with young scientists, to make his work visible, teaching his followers the lesson of creating a scientific journal which “can be truly seen” in the international scientific community. 

Many generations of students have benefited from the lessons of the mentor, and, only a few people had the opportunity of being his followers. He left them a difficult mission, that of confirming the exploration directions which they took with their mentor, being able this way, to offer identity”.

**Executive Editor** **Prof. Dr. Eng. Victor Lorin Purcarea**

